# A Careful Consideration of the Calibration Concept

**DOI:** 10.6028/jres.106.014

**Published:** 2001-04-01

**Authors:** S. D. Phillips, W. T. Estler, T. Doiron, K. R. Eberhardt, M. S. Levenson

**Affiliations:** National Institute of Standards and Technology, Gaithersburg, MD 20899-0001

**Keywords:** calibration, error, influence quantities, measurand, systematic error, uncertainty

## Abstract

This paper presents a detailed discussion of the technical aspects of the calibration process with emphasis on the definition of the measurand, the conditions under which the calibration results are valid, and the subsequent use of the calibration results in measurement uncertainty statements. The concepts of measurement uncertainty, error, systematic error, and reproducibility are also addressed as they pertain to the calibration process.

## 1. Introduction

The concept of calibration has generally been associated with statements regarding the accuracy of a standard, gauge, or measuring instrument. Although calibration typically involves many administrative, procedural, and documentary activities [1–3], in this paper we will focus on technical issues associated with measurement error and uncertainty as it relates to the calibration process. Modern metrological concepts increasingly link the topics of measurement traceability, laboratory accreditation, and quality assurance programs to the topic of measurement uncertainty. An essential component of all uncertainty budgets is the employment of calibrated gauges, standards, or instruments. It is the calibration process that transfers a reference value, usually an International System (SI) unit, to the artifact or instrument under calibration and hence establishes the “unbroken chain of comparisons” required for traceability.^1^

The ISO *International Vocabulary of Basic and General Terms in Metrology* (VIM) [4] defines calibration as follows:

Calibration (VIM-1993)—set of operations that establish, under specified conditions, the relationship between values of quantities indicated by a measuring instrument or measuring system, or values represented by a material measure or a reference material, and the corresponding values realized by standards.

Notes:

1. The result of a calibration permits either the assignment of values of measurands to the indications or the determination of corrections with respect to indications.
2. A calibration may also determine other metrological properties such as the effect of influence quantities.
3. The result of a calibration may be recorded in a document, sometimes called a calibration certificate or a calibration report.

While the body of the VIM definition is sufficiently general to accommodate nearly all calibration situations, this generality provides little guidance as to what constitutes a calibration. It is the purpose of this paper to elaborate on this topic. Our discussion will be guided primarily by the *Guide to the Expression of Uncertainty in Measurement* (GUM) [5]. We will focus our attention on three concepts associated with the VIM definition: (1) the measurand; (2) the “specified conditions” of the calibration; and (3) the “relationship” between measured or indicated values and those of reference values.

## 2. Calibration Issues

### 2.1 The Measurand

The measurand is the particular quantity subject to measurement (VIM 2.6). It is defined by a set of specifications, i.e. instructions, not a numerical value. Indeed, the measurand is an idealized concept and it may be impossible to produce an actual gauge, artifact, or instrument exactly to the specifications of the measurand. The measurand specifies the value(s) of the relevant influence quantities and these must be specified sufficiently well that any ambiguity is negligible with regard to the required accuracy of the calibration (GUM 3.3.2 and D1–D3). The more completely defined the measurand, the less uncertainty will (potentially) be associated with its realization. A completely specified definition of the measurand has associated with it a unique value and an incompletely specified measurand may have many values, each conforming to the (incompletely defined) measurand. The ambiguity associated with an incompletely defined measurand results in an uncertainty contributor that must assessed during the measurement uncertainty evaluation.

As an example of defining a measurand consider the “diameter” of a bore. The simple definition as a diameter may be sufficient for a low accuracy application, but in a high accuracy situation imperfections from a perfectly circular workpiece may be significant.^2^ Due to manufacturing imperfections, the diameter of a workpiece is incompletely defined and this can lead to “methods divergence problems” where different measuring instruments yield significantly different results. For example, when measuring a bore, a two-point diameter as measured with a micrometer,^3^ a least-squares fit diameter as measured with a coordinate measuring machine,^4^ and a maximum inscribed diameter as found using a plug gauge, will each yield a different numerical value because each measurement method realizes a different quantity permitted by the poorly defined measurand. No amount of improvement in the accuracy of these measurement methods will cause their results to converge as they are fundamentally measuring different quantities (two point, least-squares, and maximum inscribed diameters). Hence, the methods divergence problem is actually an uncertainty source associated with an incomplete definition of the measurand. A similar example is the measurement of the hardness of a material. The local hardness (microhardness) is often significantly different than the average hardness; consequently, unless a particular test location is specified, measurements at different locations can produce significantly different results.

A complete definition of the measurand will, in the general case, allow corrections to be applied for different measurement methods. For example, the calibration of a chrome-carbide gauge block using a gauge block comparator and a steel master requires the correction for the differential mechanical penetration of the probe tips since the length of the block is defined as the undeformed length.^5^ While in principle the complete definition of the measurand requires an infinite amount of information, in practice it usually contains detailed information appropriate for a particular (usually conventional) measurement method and may be significantly incomplete if alternative measurement methods are used. For example, the measurand associated with an artifact’s length might be well specified when using an instrument with mechanical contact probes (such as specifying a correction for the mechanical contact deformation), but may be less well specified when using optical or capacitance probing technologies. The use of appropriate corrections will allow convergence of the results from different measurement methods and bring them into accordance with the definition of the measurand.^6^ Hence the methods divergence problem is actually a problem with an incompletely specified measurand.

The definition of the measurand must also be sufficiently complete to avoid improper use of the calibrated artifact or instrument. For example, consider a hand held micrometer that is calibrated for measuring workpieces with flat and parallel surfaces by measuring several calibrated gauge blocks (with surfaces larger than the micrometer anvil size). This procedure does not calibrate the micrometer for measuring ball diameters because the flatness and parallelism of the anvils are unknown and are significant influence quantities for the (ball diameter) measurand.

Included in the definition of the measurand is a set of conditions that specify all the values of the influence quantities relevant to the measurand. Typically, the higher the accuracy requirements, the more extensive the list of specified influence quantities in order to have negligible uncertainty associated with the definition of the measurand. Note that definition of the measurand must address all significant conditions, i.e., influence quantities, not just environmental conditions.

### 2.2 The Specified Validity Conditions

The conditions under which the results of a calibration are valid must be stated in the calibration documentation, i.e., the calibration report. These conditions, which we will call the calibration validity conditions,^7^ include the values (or range of values) of all significant influence quantities for which the calibration results are valid. In the case of instruments, the validity conditions also include the number of measurements used to compute a result, because if repeated measurements by an instrument yield different results, then the mean (mathematical average) result will usually have a smaller uncertainty than a single result.

Generally the calibration validity conditions are either those specified in the definition of the measurand or are “extended conditions.” Typically, master gauges, artifacts, and reference standards have calibration validity conditions that are identical to the conditions specified in the definition of the measurand. For example, the results of an NIST calibrated gauge block are valid only at exactly 20 C. Although no laboratory can actually realize the conditions specified in the definition of the measurand, deviations from the validity conditions are included in the uncertainty budget of the calibration. Subsequent use of these standards, e.g., in calibrating other artifacts, will similarly not be at the validity conditions, i.e. not exactly at the conditions in the definition of the measurand. Hence the metrologist is obligated to develop an uncertainty budget which includes not only the uncertainty stated in the calibration report of the reference artifact, but also any failure to exactly realize the (measurand-defining) conditions of the reference artifact during subsequent calibrations which use the reference artifact as the “master.” Thus the uncertainty of each subsequent calibration in a traceability chain will be greater than the uncertainty of the previous calibration since the measurand-defining conditions generally cannot be fully achieved.

In contrast, some industrial calibrations involve “extended validity conditions” that are appropriate for their particular needs. These conditions may differ significantly from those that define the measurand; in particular it may include a range of influence quantities or specify a particular set of conditions that differ from those that define the measurand. For example, a factory floor worker using an instrument may not want to develop an uncertainty budget for every measurement performed. What may be desired is a calibration report that states an uncertainty under validity conditions that include the conditions of actual use. A common example is a voltmeter calibration that gives an uncertainty statement over a range of ambient temperatures. The calibration of an instrument or artifact under extended validity conditions must have its errors and uncertainties assessed over this range of conditions, or alternatively, if a sufficient model describing the behavior of the artifact or instrument exists, then the consequences of these conditions can be calculated and included in the calibration report. As with the definition of the measurand, specifying the extended validity conditions involves stating the permitted values of any influence quantity that affects the measurement. In some situations a calibration report may specify a series of uncertainty statements corresponding to a series of different validity conditions, allowing the end user to select the conditions most appropriate for the measurement.

### 2.3 The Relationship

The “relationship” between measured or indicated values and those of the reference values is a key issue with regards to calibration. The calibration process may include a wide variety of activities, including determining the mathematical relationships between influence quantities and the indications of instruments, the creation of the actual indications, e.g., the scribing of graduations onto a scale, and the adjustment of parameters to correct for known systematic effects.^8^ However, all calibrations must include a statement about the accuracy of the instrument or artifact as required by traceability. This is the relationship we will focus on in this paper. This statement may take many different forms but it describes the estimated systematic error^9^ (or the deviation from a stated nominal value^10^) and the associated uncertainty, for the specific measurand, under the validity conditions of the calibration. The values of the estimated systematic error (or deviation from nominal), together with their associated uncertainties may be expressed in a table, calibration curve, or other means of documentation.

For many instruments and artifacts, the measurement result is a continuous variable, e.g., a micrometer may measure length continuously over a zero to 50 mm interval. Since it is impossible to calibrate such an instrument or artifact for all possible values, engineering judgement must be used during the calibration process to assess the reasonable errors associated with the measurement results over the interval. What constitutes reasonable errors is best left to a standards organization or other bodies that develop performance evaluation tests specific to the particular technology relevant to the instrument or artifact under consideration. The issues of uncertainty, error, systematic error, and reproducibility are important to the topic of calibration; a discussion of these issues is presented in the Appendix.

### 2.4 Reporting Calibration Results

There are numerous different methods used to report the accuracy of calibration results. Some of the more common methods are listed below together with comments.

1. Measurement Result and Uncertainty. The best estimate of the value associated with the measurand is typically the mean of repeated measurements that have been adjusted for all needed corrections. This format is typical of artifacts or instruments that have no previous measurement history or reference value. Uncertainty statements should conform to the GUM and are usually stated (by national and international default) using a coverage factor of two (see Appendix A).
2. Deviation from Nominal or Reference Value and Uncertainty. This format is typical of artifacts that have been assigned a nominal or reference value. For example, gauge block calibrations typically report the deviation from the stated nominal length of the block and the uncertainty in this deviation. Sometimes these deviations are erroneously referred to as “corrections,” however VIM 3.15 clearly defines corrections as the negative of the estimated systematic error which involves the “true” (not nominal) value associated with the measurand.
3. Estimated Systematic Error and Uncertainty. This format is typical of instrument calibration where the reported estimated systematic error is later used to provide a correction value used in subsequent measurements. A variant of this method is to report the correction value and its uncertainty; the correction value differs in sign from the estimated systematic error and any calibration table or calibration curve should clearly indicate if the presented data is the estimated systematic error or the correction value. A special case of this method is when the estimated systematic error has been adjusted to zero. In this case it should be clearly reported that the estimated systematic error is zero and the remaining uncertainty stated.
4. Metrological Requirements. This format is typical of industrial calibrations where some requirement has been established, typically a “maximum permissible error” (MPE) for instruments or a maximum deviation from nominal for artifacts. For example, a calibration of a hand-held micrometer could be required to demonstrate conformance with a stated MPE value. Similarly a calibration of a gauge block could be required to demonstrate that the deviations from nominal be less than some stated value provided by a grade classification in order for the block to be in conformance with that grade. When demonstrating conformance to a metrological requirement, the decision rule should be stated in the calibration report.^11^ (A decision rule clearly states how measurement uncertainty will be addressed when demonstrating conformance according to specifications.)

## 3. Subsequent Measurement Uncertainty Statements

The results of a calibration describe the value and uncertainty associated with our knowledge of a specific measurand under specified validity conditions for an artifact or instrument. Typically, the artifact or instrument is used in subsequent measurements that are not calibrations. A traceable measurement requires both an unbroken chain of comparisons back to a reference value (typically a SI unit) and also an uncertainty statement. It is the use of a calibrated instrument or artifact in a measurement that provides the unbroken chain of comparisons back to the reference value. However, frequently the uncertainty statement provided by the calibration is insufficient for the subsequent measurement under consideration since the validity conditions of the calibration do not include those of the subsequent use. In some cases involving complex instruments, the measurand of the subsequent measurement may be significantly different from the measurand of the master used to calibrate the instrument. Consequently, it is up to the end user or metrologist to create an uncertainty statement for the measurement of interest. We now consider the relationship between calibration results and subsequent measurements.

The definition of the measurand of the calibrated instrument or artifact includes a stated set of conditions for all influence quantities. Similarly, the calibration results are valid for a specified set of validity conditions which may (or may not) be the same as the conditions in the measurand definition. We now introduce the measurement conditions that specify the values of the influence quantities that prevail during the subsequent measurements using the calibrated instrument or artifact. Two cases are possible:

1. Measurement conditions are included in the calibration validity conditions. In the case of a calibration with extended validity conditions, it is possible that the measurement conditions are contained within the calibration validity conditions. In this case the measurement uncertainty statement can be obtained directly from the calibration report. Additionally the calibration report may contain sufficient information to correct the measurement result for the estimated systematic error associated with the artifact or instrument.
2. Measurement conditions different from calibration validity conditions. In this case, the measurement conditions are not contained in the calibration validity conditions. This will always be the case for an artifact or instrument with calibration validity conditions specified as the conditions in the definition of the measurand, i.e., not extended validity conditions. Alternatively, this may also occur when the calibration’s extended validity conditions do not fully include the measurement conditions. In these cases the information contained in the calibration report is not sufficient and it is necessary to develop an uncertainty budget for the subsequent measurement. In some cases developing the uncertainty budget may be quite simple, e.g., an instrument that is calibrated under a set of extended validity conditions that includes measuring steel artifacts is now used to measure aluminum artifacts; this new measurement condition might be easily be taken into account since the properties of materials are generally well known. In other cases the uncertainty budget may be very difficult to develop, e.g., an instrument with a complex dependence on environmental conditions, is used in environmental conditions significantly different from those of the calibration validity conditions. To create a measurement uncertainty statement for this case, there must be an acceptable procedure to assess the change of the estimated systematic error and the uncertainty from the calibration validity conditions to the measurement conditions. Such an evaluation will always increase the measurement uncertainty because it will add new corrections for systematic effects, together with their uncertainties associated with the measurement conditions. Some methods to evaluate these effects include:
  - Guidance, such as evaluation procedures, provided in the calibration report.
  - Instrument performance specifications provided by the manufacturer.
  - Mathematical/physical model of the measurement process. Such a model provides a functional relationship between the value of the measurand indicated by the instrument and the relevant condition parameters. (Typical condition parameters might be temperature, workpiece thermal expansion coefficient, etc.)
  - Heuristic plausibility model argued from an expert Type B perspective. Acceptance of such an evaluation will depend strongly on the perceived qualifications of the expert.

## 4. Summary

We have described several of the technical issues associated with the calibration process. The distinction between the measurand conditions, the calibration validity conditions (not to be confused with the conditions prevailing at the time of the calibration), and the conditions of subsequent measurements are emphasized. The use of calibrated instruments or artifacts in traceable subsequent measurements will require the development of their own uncertainty statement if the conditions of measurement are outside the calibration validity conditions.

## Figures and Tables

**Fig. 1:**
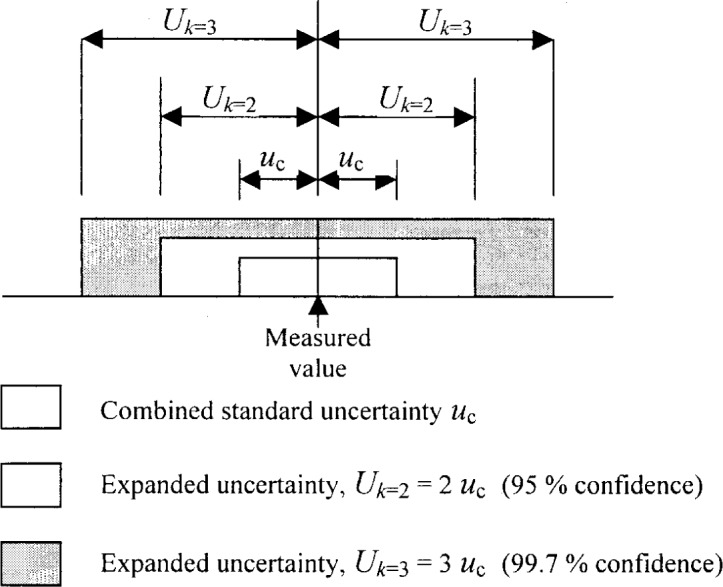
Diagram illustrating the distinction between combined and expanded uncertainty. The level of confidence associated with each of the uncertainty intervals shown assumes a Gaussian probability distribution for the possible values of the measurand. Note: the stated level of confidence will only be achieved if the uncertainty contributors are well evaluated and the effective degrees of freedom is large.

**Fig. 2:**
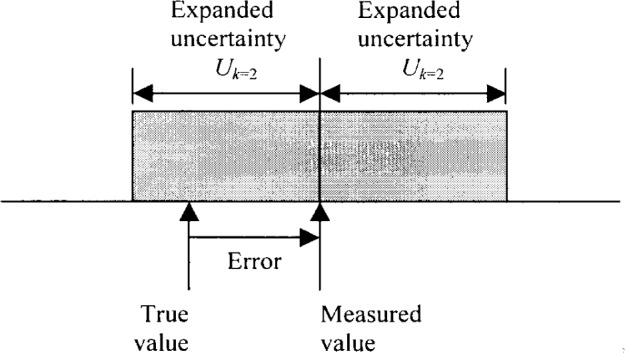
Illustration of the difference between the measurement error and the measurement uncertainty. Since the “true value” of the measurand is never exactly known, the error can only be estimated.

**Fig. 3:**
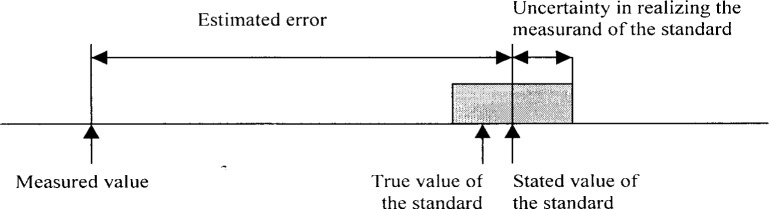
Illustration of the estimated measurement error of a standard during a calibration procedure.

**Fig. 4:**
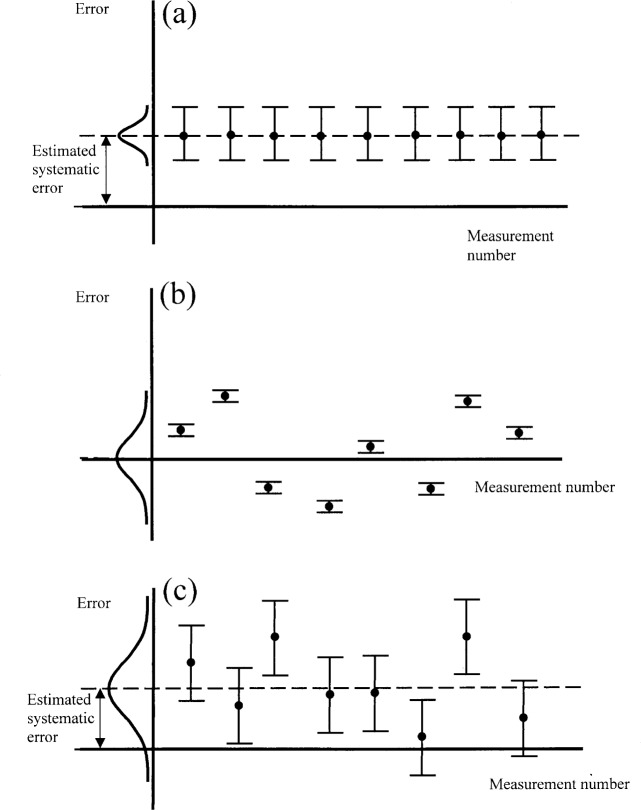
A schematic diagram depicting the distribution of potential errors; it is assumed that the repeated measurements occurred over a sufficiently long time to include all reproducibility effects. (a) Repeated measurements with excellent reproducibility, a large estimated systematic error, and a significant uncertainty associated with the realization of the measurand as represented by the large “uncertainty bars”; (b) repeated measurements with no estimated systematic error, small uncertainty associated with the realization of the measurand, and poor reproducibility as represented by the large spread in the data points; (c) the typical case combining estimated systematic error, uncertainty associated with realizing the measurand, and poor reproducibility.

## Footnotes

1 Even calibrations that use “self-calibration” methods, e.g., straightedge reversal using an indicator, require uncertainty statements since the uncertainty of the indicator must be assessed.

2 For this reason, some standards such as ASME Y14.5 have further defined the diameter of a bore to be the maximum inscribed diameter.

3 A “two-point diameter” is also an ambiguous measurand since different cross sections will in general yield different two-point diameters.

4 A least-squares diameter fit has an unambiguous value when computed from an infinite number of points; the effect of finite sampling must be included as a measurement uncertainty source.

5 The calibration of gauge blocks made of the same material as the master generally do not require a penetration correction since the deformation is the same on both blocks and hence cancels out.

6 In some cases a metrologist will deliberately choose (for economy or convenience) to measure a related quantity that differs from the measurand, e.g., a least-squares diameter instead of a maximum inscribed diameter. In this case a estimated systematic error results, which must either be corrected or accounted for in the uncertainty statement of the measurement.

7 We use the term calibration validity conditions in order to avoid confusion with the conditions that happen to prevail at the time of the calibration.

8 Some accreditation programs require a calibration before and after adjustments are performed so that the metrological history of the instrument or artifact is maintained.

9 We use the term systematic error in the VIM sense, as the mean error of an infinite number of measurements.

10 For reference artifacts, e.g. gauge blocks, it is usually more useful to state the “deviation from the nominal value.”

11 Some accreditation programs may require the use of a specific decision rule in order for the measurement to be considered a calibration.

## 5. Appendix A. Uncertainty, Error, Systematic Error, and Reproducibility

*Uncertainty* of measurement, in its broadest sense, means doubt about the validity of a measurement result (GUM 2.2.1). *The ISO International Vocabulary of Basic and General Terms in Metrology* (VIM) defines uncertainty as a parameter, associated with the result of a measurement, that characterizes the dispersion of the values that could be reasonably attributed to the measurand (VIM 3.9). (The “measurand” is the specific quantity being measured.) It is noteworthy to point out, firstly, that an uncertainty statement is associated with a measurement result, not with the measurement instrument (although the instrument is an uncertainty contributor), and secondly, that measurement uncertainty is associated with a specific measurand and, in general, different measurands may have different uncertainty statements even if they are measured with the same instrument. The modern method of expressing measurement uncertainty involves summarizing the combined effects of all uncertainty sources in terms of a single quantity, known as the combined standard uncertainty *u*_c_. In most industrial settings, measurement uncertainty is expressed as a multiple (given by the coverage factor *k*) of the combined standard uncertainty, yielding the expanded uncertainty *U*, so that *U* = *k u*_c_. The expanded uncertainty can be used to define an interval, *y* ± *U* where *y* is the result of a measurement, that may be expected to encompass a large fraction of the distribution of values that could be reasonably attributed to the measurand (GUM 2.3.5). Furthermore, the expanded uncertainty is often associated with a level of confidence through the coverage factor; the typical default value of the coverage factor is two (*U_k_*_= 2_ = 2*u*_c_), which is generally considered to imply a level of confidence of approximately 95 %; see Fig. 1. The specific level of confidence requires assumptions about the probability distribution that is characterized by the measurement result and its combined standard uncertainty. However, using the default coverage factor of two and assuming a 95 % confidence is usually a reasonable approximation provided the effective degrees of freedom is reasonably large, e.g., ≥20 and the uncertainty contributors have been well evaluated. A 95 % level of confidence implies that 95 % of the values that can be reasonably attributed to the measurand lie within an uncertainty interval of *U_k_*_= 2_ that is centered on the measurement result.

The *error* in a measurement result is defined as the measured value minus the “true value” of a measurand (VIM 3.10); see Fig. 2. Strictly speaking, the error of a measurement result is never exactly known since the value of a measurand is never exactly known. However, useful estimates of an error are possible when the uncertainty in the error is small relative to the magnitude of the error. Hence errors can only be estimated when performing a measurement of (or comparison to) a standard that has a previously assigned value so that an independent estimate of the “true value” of the measurand is available.^12^ It is worth reiterating that when estimating an error the measured value is whatever number is reported by the measurement system, and as far as determining the error of that measurement is concerned, the measured value is an exact, well-defined, value see Fig. 3.^13^

The uncertainty of a standard used to realize a measurand includes not only the uncertainty documented in its calibration report but also any additional uncertainty associated with the conditions the prevail at the time it is used in the calibration. For example, a gauge block with a length specified at 20 °C (the measurand) may be used to calibrate an instrument at 21 °C. After the correction for the thermal expansion of the block, there remains the uncertainty associated with the measurement temperature differing from the measurand defined conditions. This additional uncertainty must be combined with the uncertainty associated with the standard stated at 20 °C. The effect of any influence quantity present during the calibration that degrades the accuracy of the reference standard must be included in the uncertainty associated with the realization of the measurand during the calibration.

For general workpiece measurements, the measurand is an attribute of the workpiece and its “true value” is unknown (hence the point of making the measurement), and therefore the measurement error is similarly unknown. Thus, for most workpiece measurements, it is incorrect to speak of the measurement error^14^ and the appropriate term for the workpiece measurement situation is measurement uncertainty.

*Systematic Error* is the (mathematical) expectation value of the error. It can be estimated as the mean error in the reported value of a measuring instrument or of an artifact. Similar to the case of error, the systematic error is never exactly known because we never know the “true value” and we cannot perform an infinite number of measurements of a standard to produce the expectation value. The estimated systematic error may be determined from the mean of a series of repeated measurements or as a calculated value corresponding to a known systematic effect. Figure 4(a) illustrates a series of measurements that have good reproducibility but contain a large estimated systematic error in addition to a significant uncertainty associated with the realization of the measurand. As previously described, realization of the measurand includes the uncertainty associated with the reference standard under the conditions employed during the calibration.

The *reproducibility* of a measurement is “the closeness of agreement between results of measurements of the same measurand carried out under changed conditions of measurement” (VIM 3.7). For a calibration, the sources of the changing conditions may correspond to variations in any of the influence quantities in the definition of the measurand (some of these changes might be manifested by changing the metrologist and the measuring instrument). While each of the observed estimated errors could have a small uncertainty (because the uncertainty associated with realizing the measurand is small), the measurement uncertainty may be large due to the reproducibility; see Fig. 4(b).

In a typical calibration, the measurement uncertainty is usually a combination of the uncertainty associated with the standard (i.e., the realization of the measurand), that associated with measurement reproducibility, and other static effects (e.g., a sensor may have an unkown fixed offset from its calibrated value and consequently this effect may not appear in the reproducibility evaluation^15^); see Fig. 4(c).
